# Bats flying through a Y-maze are visually attracted to wind turbine surfaces

**DOI:** 10.1098/rsbl.2025.0242

**Published:** 2025-08-13

**Authors:** Kristin A. Jonasson, Aaron J. Corcoran, Laura Dempsey, Theodore J. Weller, Jeff Clerc

**Affiliations:** ^1^ Independent Researcher, USA; ^2^ University of Colorado Colorado Springs, Colorado Springs, CO, USA; ^3^ National Renewable Energy Laboratory, Golden, CO, USA; ^4^ USDA Forest Service Pacific Southwest Research Station, Arcata, CA, USA

**Keywords:** sensory biology, sensory pollutant, ecological trap, wind energy, hoary bat (*Lasiurus cinereus*), silver-haired bat (*Lasionycteris noctivagans*)

## Abstract

Wind energy’s rapid expansion has led to unintended consequences for wildlife, with migratory bats among the species most at risk. The behavioural mechanisms underlying collisions remain poorly understood, but one hypothesis is that bats are attracted to wind turbine structures. Vision is important to bat orientation and obstacle avoidance, yet it has been relatively understudied in the context of bat–turbine interactions. We hypothesize that light reflected off turbine surfaces could attract bats, acting as a sensory pollutant that may increase collision risk. To test whether reflective turbine surfaces elicit attraction, we flew 242 *Lasiurus cinereus* and 154 *Lasionycteris noctivagans* through Y-maze assays. Bats were at least twice as likely to fly towards white turbine blade sections compared to less reflective black ones. This attraction intensified when the alternative exit was a dark, empty flyway, with 74% of *L. cinereus* and 97% of *L. noctivagans* flying towards the white turbine blade. These findings provide evidence that visual sensory pollutants could underlie bat–turbine interactions, and if so, wind turbines could be ecological traps.

## 1. Introduction

The expansion of human activities into the aerosphere has led to unexpected interactions with wildlife [1]. Among these, collisions with industrial wind turbines have emerged as a threat to bat populations [2–5]. There is widespread speculation that bats are drawn to turbines, perceiving them as potential roosting, foraging or lekking sites [6,7], or that turbine stimuli may disrupt orientation [8]. Evidence of attraction remains correlative, with higher bat activity near turbines [9] and after construction of some wind energy facilities [10]. Support for the attraction hypothesis comes from video of bats that seem to linger in turbines’ airspace, repeatedly approaching the structures—flying up the monopole while ‘skimming’ its surface or ‘chasing’ blades [11–14]. Such interactions may lead to fatal collisions [11,13,14]. In addition to generating power, turbines produce stimuli perceptible to bats: acoustic noise, turbulent airflow and visual cues from structures and lighting [8]. These sensory pollutants may interfere with animal perception and behaviour by misleading, distracting or masking natural cues [15,16]. Ecological traps occur when animals' responses to cues, once adaptive or neutral, lead to higher mortality in human-modified landscapes [17,18]. If bats are attracted to wind turbines and experience higher mortality as a result, then wind turbines may act as ecological traps.

Visual stimuli are strong candidates for attractants given their importance to bat orientation [19,20], navigation [21–28] and obstacle avoidance [29,30]. Further, sight often takes precedence over other senses (i.e. touch and echolocation) in decisions of where bats fly [19,31–33]. Attraction to artificial light at night (ALAN) is a widespread behaviour, creating ecological traps for insects [34], fishes [35], reptiles [36] and birds [37,38]. Bat responses to ALAN vary by species and are influenced by morphology and flight behaviour. Fast-flying, open-air foragers appear more susceptible to collisions with turbines [39] and bats with these attributes are generally tolerant of ALAN [40]. Although some bats are known to forage on insect aggregations around ALAN [40], increased bat activity near ALAN can occur without evidence of elevated feeding [41,42]. ALAN may also disrupt bat orientation and navigation, as suggested by collisions of migratory bats with tall, lit structures [43–45]. Research has explored bat attraction to red obstruction lights equipped on some turbines (reviewed in [8]), but the potential for turbine surfaces themselves to act as attractants has not been examined.

We hypothesized that ambient light reflected off white turbine surfaces may elicit behavioural responses in bats, given the sensitivity of bat vision to dim light [46,47] and the high reflectivity of these surfaces [48]. White surfaces can disrupt bat flight behaviour and obstacle avoidance, occasionally resulting in collisions [49,50]. Where humans see the distinct shape of turbines, bats have less visual acuity and might perceive these exceptionally large, white structures as brighter regions of the sky [8]. Bats are thought to perceive brighter areas in their visual field as the direction of the open sky [19,32,51], which may result in bats mistaking turbines for unobstructed airspace. Videography of bats closely approaching turbine surfaces (<5 m), sometimes repeatedly [11–13], resembles behaviours described in early studies on the use of vision by bats, which established that attempts to fly through backlit windows were vision-mediated [31,32]. Although collisions with stationary turbine components (i.e. monopoles, nacelles) are unlikely to harm bats, approaching them brings them into close proximity of the rotor-swept zone where blade strikes occur. This is concerning given that close approaches to stationary turbine components occur more frequently than all other flight behaviours combined (e.g. blade approaches, hovering, pass) [12,13]. To test our hypothesis, we used a Y-maze experiment to determine whether dimly lit turbine surfaces can attract two of the migratory bat species most impacted by wind energy in North America—hoary bats (*Lasiurus cinereus*) and silver-haired bats (*Lasionycteris noctivagans*).

## 2. Material and methods

### (a) Capture and Y-maze assays

**Figure 1.**
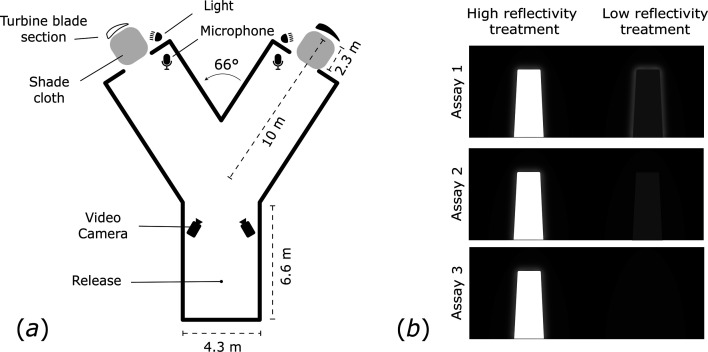
(*a*) Experimental Y-maze apparatus, used to test the orientation preferences of *Lasiurus cinereus* and *Lasionycteris noctivagans* for sections of decommissioned wind turbine surfaces with varying reflectance. The height of the apparatus was 2.35 m throughout. (*b*) Point-of-view of the choices presented to bats during the assays. High-reflectivity treatment: a white turbine blade lit with artificial moonlight; low-reflectivity treatments: a black turbine blade lit with artificial moonlight (assay 1: black lit), an unlit black turbine blade (assay 2: black unlit) or an unlit exit with no turbine blade (assay 3: empty unlit).

We captured bats in mist nets along the Bull Creek riparian corridor in Humboldt Redwoods State Park, California, USA, between 15 September and 13 October 2024. Bats were processed, marked with PIT tags (*L. cinereus*) or forearm bands (*L. noctivagans*), held in individually numbered cloth bags within a cooler, and transported <3 km to the Y-maze. Bat capture and handling were conducted under California Department of Fish and Wildlife scientific collections permit (no. S-230490001-23057-001) and approved by the USDA Forest Service Research and Development Institutional Animal Care and Use Committee (no. 2024-010). Once in the Y-maze, bats were removed from holding bags in darkness and allowed to fly off a gloved hand when ready. Most individuals flew within 5 s. Their choices were scored in real time by an observer watching the live feed of an infrared video system (described below) and later independently verified by a second observer in the lab watching slowed-down videos. Bats chose between a high- and low-reflectivity treatment in each of three Y-maze assays (figure 1). In all assays, one arm featured a high-reflectivity treatment: a section of a decommissioned white turbine blade lit with artificial moonlight. The other arm presented one of the following progressively lower reflectivity treatments: a black turbine blade lit with artificial moonlight (*assay 1: black lit*), an unlit black turbine blade (*assay 2: black unlit*) or an unlit arm with no turbine blade (*assay 3: empty unlit*). After exiting the Y-maze, bats flew away into the environment. To avoid directional bias, we switched the treatment condition between the right and left Y-maze arms such that no more than 10 *L*. *cinereus* or 10 *L*. *noctivagan*s completed the assay before the switch. Upon trial completion, the holding bag number was read to identify each bat. As bats have been PIT-tagged at this site over several years, it would have been logistically challenging to identify and exclude bats that had flown the Y-maze previously. Of the 25 *L*. *cinereus* flown more than once, 21 participated in two assays and four participated in all three assays; no bat flew the same assay more than once. Bats that flew in multiple assays were tested on average 12 days apart (min = 5 days, max = 28 days). Bats that flew in more than one assay did not differ statistically in the preference within the Y-maze compared to bats being run through an assay for the first time (Fisher’s exact test, *p* = 0.21).

### (b) Apparatus

We constructed the Y-maze by custom-fabricating a steel frame caterpillar tunnel hoop house (Farmers Friend, Centerville, TN, USA) (figure 1). We lined the structure with 90% shade cloth (Tunnel Vision Hoops LLC, Shaker Heights, OH, USA) to reduce the intensity of echoes and covered the exterior with silage tarp to black out ambient light. We placed sections of 90% shade cloth on the ground between the turbine blade section and the Y-maze arm exits to minimize light reflecting off the ground. Two turbine blade sections of identical size and shape (1.8 m height, 1.0 m base width and 0.8 m top width) were cut from the rotor of a Northwind 100C turbine (Northern Power Systems, VT, USA). We selected turbine blades as experimental surfaces because they are the largest structures logistically feasible for use in our assay. At a distance of 10 m, the turbine blade subtended an angular width of 5°, equivalent to the width of a 7 m diameter turbine monopole viewed from 80 m away. The high-reflectance surfaces were coated with industry-standard glossy white paint, while low-reflectance surfaces were painted with exterior latex matte black (ACRI-SHIELD® MAX). Turbine blades were indirectly illuminated with Telelumen Octa Light Players (Telelumen LLC, CA, USA), set to replicate moonlight spectra (electronic supplementary material, figure S1). We measured reflected light intensities at the maze exit using a light meter (Extech LT300; Industrial Electronics Inc., Knoxville, TN, USA) held perpendicular to the reflective surface (white lit: 0.10 lx; black lit: 0.06 lx; black unlit: 0.03 lx; empty unlit: <0.01 lx). We filmed bats in the Y-maze using two Ace acA2000-50gc infrared video cameras (Basler, Highland, IL, USA) recording at 30 frames per second and 1024 × 524 pixel resolution. Infrared illumination was provided by two Raymax 200 infrared illuminators (Raytec, Ashington, UK). Video was captured on a laptop computer running custom acquisition software coded in MATLAB v. 2024 (MathWorks, Natick, MA, USA). Ultrasound was recorded using a two-channel USGH 416 h recording unit (Avisoft Bioacoustics, Glienicke, Germany) and Avisoft CM16/CMPA microphones. We placed cameras to view the two exit arms of the Y-maze and microphones were placed just inside the exits.

### (c) Acoustic analysis

We processed all audio files automatically using custom MATLAB v. 2024 (MathWorks) scripts to extract the first series of echolocation calls after each bat release from the microphone on the side where the bat flew. We followed the protocols described in a previous study of *L. cinereus* echolocation [52] to extract call duration, pulse interval and peak frequency. In some cases, call intensities were not sufficiently high in the recordings to be extracted automatically but were instead reviewed manually. These recordings were not included in our descriptive statistics of bat echolocation parameters but were used to confirm whether bats were echolocating.

### (d) Statistical analysis

We tested for a right-side/left-side bias using Fisher’s exact test on the proportion of bats that flew towards the high-reflectivity treatment when it was on the right versus when it was on the left. This, and all other tests, was repeated for each assay and species. We used binomial tests to determine if bats flew towards the high- or low-reflectivity treatment more often than chance for each of the three assays. We tested for species differences among assays using Fisher’s exact test. All statistics were conducted in R Studio v. 2024.04.2 [53] using an alpha value of 0.05.

## 3. Results

**Figure 2.**
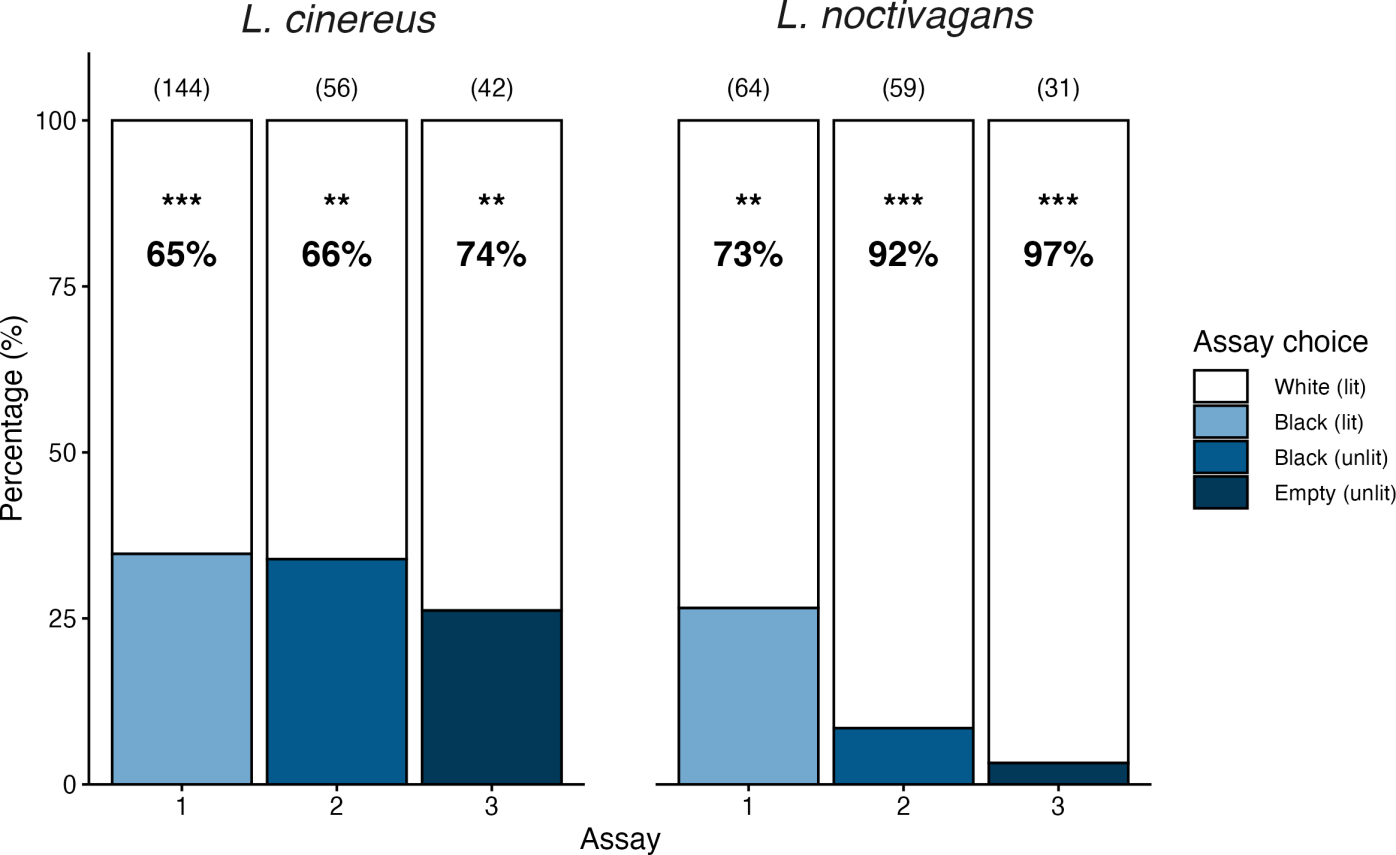
Percentage of *Lasiurus cinereus* and *Lasionycteris noctivagans* selecting the high-reflectivity (white) turbine blade dimly lit with artificial moonlight versus the low-reflectivity (black) turbine blade or empty runway in a Y-maze. Sample size shown in parentheses. **p < 0.01 and ***p < 0.001 indicate significant differences from 50% (no preference).

*Lasiurus cinereus* and *L. noctivagans* both showed a preference to fly towards the high-reflectivity treatment (white turbine) over the less-reflective alternative in all three assays (figure 2; table 1; electronic supplementary material, video S1). As the brightness of the less reflective alternative decreased from experiments one through three, the proportion of bats exiting towards the white, dimly illuminated turbine surface increased. In assays 1 and 2, *L. noctivagans* were more likely to approach the black turbine surface when it was dimly lit versus unlit (Fisher’s exact test, *p* = 0.01), whereas *L. cinereus* did not respond differently to these two treatments (Fisher’s exact test, *p* = 1). *L. noctivagans* had a stronger response than *L. cinereus* in assays 2 and 3, but not in assay 1 (Fisher’s exact test, assay 1, odds ratio = 0.68, *p* = 0.26; assay 2, odds ratio = 0.18, *p* = 0.001; assay 3, odds ratio = 0.10, *p* = 0.01).

We observed no collisions between bats and turbine surfaces during our trials. We did not observe a left–right side bias in the Y-maze for either *L. cinereus* (Fisher’s exact test; *n* = 251 trials; odds ratio = 0.90; *p* = 0.78) or *L. noctivagans* (*n* = 168; odds ratio = 0.72; *p* = 0.5). In all trials, bats emitted echolocation calls typical of a cluttered environment (*L. cinereus*: *n* = 192 trials, mean ± standard deviation, pulse interval = 41 ± 15 ms; peak frequency = 41.5 ± 28.8 kHz; pulse duration = 1.99 ± 0.5 ms; *L. noctivagans*: *n* = 97 trials, pulse interval = 37.9 ± 17.3 ms; peak frequency = 37.5 ± 31.1 kHz; pulse duration = 1.96 ± 0.64 ms). No statistical differences in any call parameter were observed for either species approaching the dim-lit white turbine surface versus any of the other three treatments (ANOVA, *p* > 0.05).

**Table 1.** Percentage of *Lasiurus cinereus* and *Lasionycteris noctivagans* selecting the high-reflectivity (white) turbine blade dimly lit with artificial moonlight versus the low-reflectivity (black) turbine blade or empty runway in a Y-maze. Proportions >50% indicate a preference for the high-reflectivity treatment.

Y-maze choices			*Lasiurus cinereus*				*Lasionycteris noctivagans*			
assay	high-reflectivity	low-reflectivity	%	*N*	ratio	*p*‐value	%	*N*	ratio	*p*‐value
1	white lit/obstructed	black lit/obstructed	65	144	1.88	<0.0001	73	64	2.09	0.002
2	white lit/obstructed	black unlit/obstructed	66	56	1.95	0.011	92	59	6.11	<0.0001
3	white lit/obstructed	empty unlit/open	74	42	2.82	0.001	97	31	15.5	<0.0001
total			67	242	2.03	<0.0001	85	154	3.94	<0.0001

## 4. Discussion

Our experiments demonstrate that both *L. cinereus* and *L. noctivagans* are attracted to ambient light reflected from wind turbine surfaces. We found that bats selected the exit with the white turbine surfaces at least twice as often as the less reflective exit. Bats showed the strongest preference for the white turbine exit when the alternative was an opening with no turbine blade (*L. cinereus* 2.9 × more, *L. noctivagans* 15.5 × more), as would be expected if bats prioritized vision over echolocation. The hypothesis that bats are attracted to some aspect of turbines has been widely speculated [6–8,12], and our study provides evidence for a visual mechanism of attraction. Our work identifies a link between a sensory pollutant—reflective turbine surfaces—and a risky behaviour: approaching these surfaces. Approaching turbine surfaces is not inherently dangerous—bat fatalities are absent at non-operational turbines [54,55], but near operating turbines, bats risk collision with fast-moving blades. We predict that bats may be visually attracted to turbines when they occupy 5° of their visual field, which corresponds to 80 m away for many industrial turbines. Bats in our study flew through a confined testing arena with a binary choice, whereas bats in the aerosphere have more time and more options for considering whether to approach wind turbines. Turbine monopoles could have a stronger attractive effect as they are very large, and as bats approach, turbine surfaces will reflect greater amounts of ambient light and comprise a relatively larger proportion of their visual field. Whether the attraction observed in the Y-maze extends to free-flying bats interacting with full-scale wind turbines in open air remains unknown and will require further studies.

Our study was not designed to distinguish bats’ motivations for selecting the exit with the highly reflective cue. Rigorously testing the mechanisms of attraction to ALAN is challenging and has only recently been resolved for flying insects [56]. A proclivity to orient towards light when seeking an exit is likely an ancestral trait, occurring in at least five major families of bats, and is thought to be useful in exiting caves [19,57]. Most studies of this behaviour have tested cavity-roosting bats crawling through a small Y-maze [19,51,57,58]. Until now, it was unclear whether flying, foliage-roosting bats like *L. cinereus* would exhibit similar attraction to light as cavity-roosting bats or if light reflected off turbine surfaces would be sufficient to elicit this behaviour. We hypothesize that bats in our study and those interacting with turbine structures [11–14] may be exploring areas that they perceive as more open to the sky. Two potential criticisms warrant consideration. First, wind turbines are not located in caves. There is no reason why bats’ use of dim light as an orientation cue should be limited to roost scenarios. This is exemplified by instances of free-ranging bats colliding with artificially lit surfaces, e.g. white trailers, backlit windows and lighthouses [43,45,50]. Second, some early publications use the term ‘visual escape response,’ [19,32,51] to describe this behaviour, which implies a physiological state of fear or stress, but bats are not necessarily trying to ‘escape’ when in the vicinity of turbines. However, movement towards lit surfaces is still observed in less stressful scenarios, i.e. long-term captive bats tested in familiar spaces [19]. Further, it is not expected that stress is necessary to integrate information from a neutral orientation cue routinely used to depart roosts. Consequently, neither restricted airspace nor stress is required for this mechanism of attraction. Our study does not preclude the alternative hypothesis that bats approaching turbines from greater distances (≳100 m) mistake light reflected from turbine surfaces for celestial orientation cues [20]. We consider it unlikely that bats were selecting the white turbine treatment for prospective foraging. Although insects can aggregate around turbines [59], migratory bats at this site and time of year produce relatively little guano [60] and do not appear to be foraging intensely.

Our Y-maze experiment provides insight into patterns of bat behaviour and mortality at wind facilities. Cryan *et al.* [12] detected bats within the rotor-swept zone of turbines more frequently during periods of bright moon illumination and suggested that vision plays an important role in bat attraction to turbines. Smallwood & Bell [61] linked increased bat fatalities on nights with greater moon illumination with increases in risky flight behaviours (3.6×) and near misses (8×). To our knowledge, only one study has examined the effect of lunar illumination on nightly mortality at the species level; Baerwald & Barclay [62] found *L. noctivagans* mortality was significantly higher on nights with greater moon illumination, whereas *L. cinereus* mortality showed no such pattern. This does not negate the potential role of vision in bat attraction to turbines. Instead, it suggests that variation in moonlight intensity may not significantly affect the strength of attraction for some species. Although our study did not explicitly replicate light intensities associated with different moon phases, our low-reflectivity treatments progressively reduced the amount of reflected light. Although both species were attracted to white turbine surfaces, reducing light intensity of the alternative exit had no effect on *L. cinereus* but significantly decreased *L. noctivagans* selection of black surfaces. Nevertheless, we do not over-interpret the relative strength of *L. cinereus* versus *L. noctivagans* attraction to higher-reflectance treatments, as the smaller size and lower wing loading of *L. noctivagans* may have made them more capable of manoeuvring and exerting stronger choice within the Y-maze.

Several factors might elevate bat susceptibility to wind turbines as potential ecological traps. Visual cues are likely given more weight than echolocation when flying in the open airspace of wind facilities as vision has greater range and field of view and is not masked by wind noise. The airspace near turbines is likely a challenging sensory environment where distracting stimuli could impair decision-making [8]. Finally, bats buffeted or tumbled by the wake of moving blades could be momentarily disoriented [14]. To human observers, sensory ecological traps can produce baffling behavioural mismatches (e.g. [18,63,64]). Robertson *et al.* [18] proposed that novel cues are especially likely to trap animals when they resemble historically good choices that are reliable indicators of option quality, and there is low risk to investigating poor options. Light reflected off turbine surfaces meets these criteria: large reflective surfaces could resemble cues associated with open sky (discussed above); historically, light at night was likely a reliable indicator of the sky; and the cost of investigating lit surfaces is typically low as bats can veer away or recover from collisions [31,50] without injury [63]. Our study provides support for the hypothesis that light reflected from wind turbine surfaces may underlie bats maladaptive behaviours near wind turbines. Further research is needed to fully understand the effects of reflective turbine surface on bats and should include (i) quantifying the spatial scales at which visual cues from turbines influence behaviour of free-flying bats; (ii) identifying which wavelengths of light elicit the strongest response in bats; and (iii) re-examining datasets of nightly bat fatalities at wind facilities for species-specific responses to changes in ambient light levels. These insights could inform the development of mitigation strategies to reduce bat attraction and mortality at wind energy facilities.

## Data Availability

Data and analysis code supporting this study are available in the electronic supplementary material. Supplementary material is available online [65].
